# Computational data as the fuel for AI in chemistry

**DOI:** 10.1039/d6sc03659g

**Published:** 2026-09-09

**Authors:** Ross James Urquhart, Tell Tuttle

**Affiliations:** a Department of Pure and Applied Chemistry, University of Strathclyde 295 Cathedral Street Glasgow G1 1XL UK tell.tuttle@strath.ac.uk ross.urquhart@strath.ac.uk

## Abstract

The rapid growth of machine learning and generative AI in the chemical sciences has placed increasing demands on the data used to train and evaluate these models. Although algorithmic advances have attracted considerable attention, the quality, consistency, coverage and accessibility of chemical data remain major constraints on AI-driven discovery. This perspective argues that computational data should be regarded not merely as a complement to experimental data, but as deliberately designed, domain-specific infrastructure for chemical AI. This does not require exhaustive coverage of chemical space: the distinctive value of computational methods lies in their ability to generate reproducible, consistently labelled data systematically within selected regions of chemical and configurational space. Through case studies in neural-network potentials and computational peptide design, we examine how the selection, sampling, fidelity and scale of computational datasets define model capabilities and domains of applicability. We then consider how computational and experimental data can be combined, with computation enabling scalable, targeted data generation and experiment providing physical grounding and validation. We conclude by identifying priorities for deliberate dataset design, FAIR data practices and open benchmarking in chemical AI.

## Abstract 2

Computational data provides the scalable, systematic foundation needed to fuel AI in chemistry, enabling more reliable predictive and generative models through deliberate dataset design, hybrid validation, and FAIR benchmarks.

## Graphical abstract

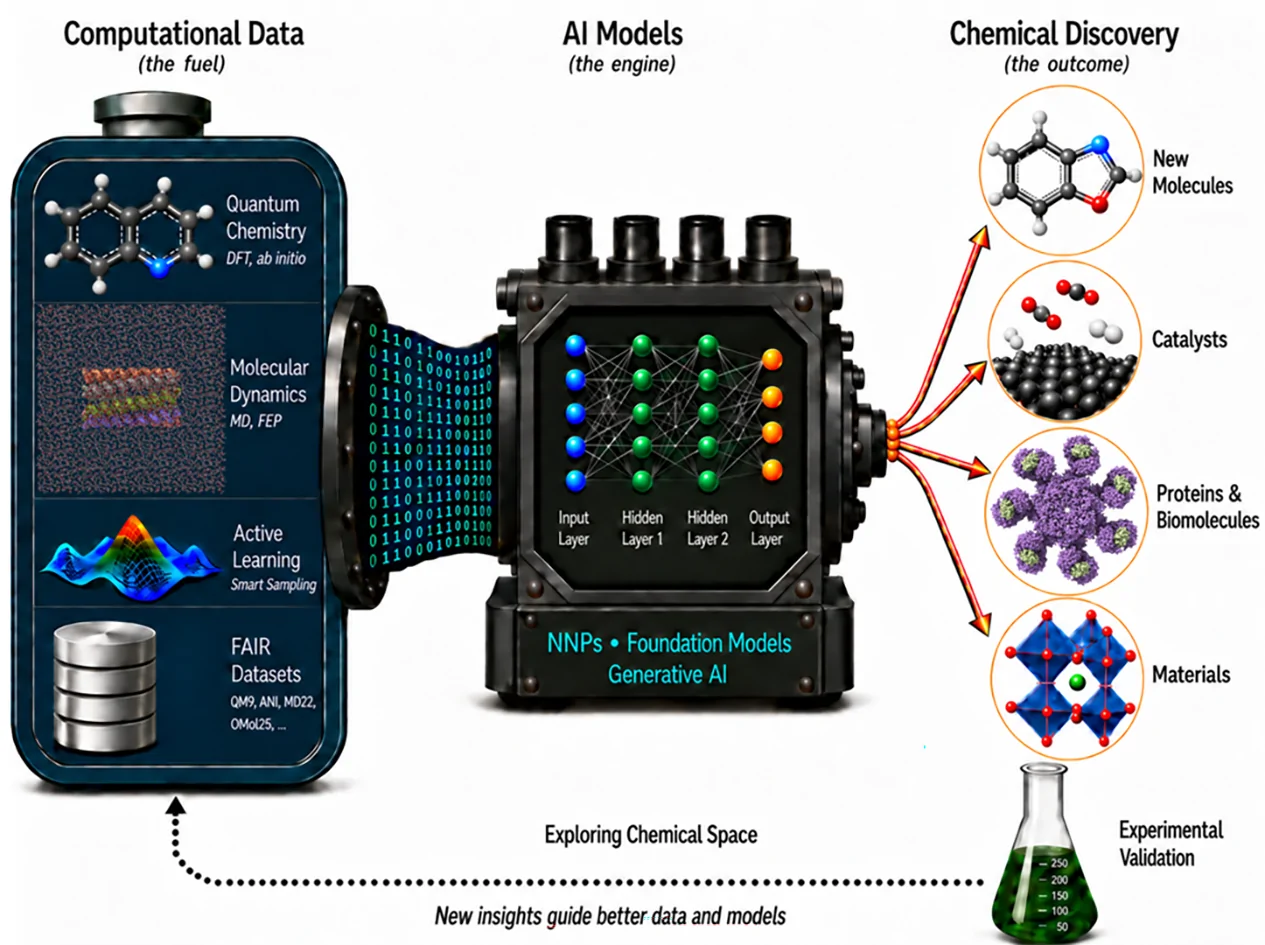

## The data foundations of AI in chemistry

Chemistry sits at an inflection point. The algorithms driving machine learning (ML) and generative AI in the chemical sciences have matured rapidly, but what has not kept pace is the data infrastructure upon which those algorithms depend. Here, domain-specific data infrastructure refers to the datasets, generation protocols, metadata, benchmarks and access mechanisms required to support reproducible model training and evaluation within a defined chemical or configurational domain. Indeed, most chemical ML failures are data design failures, not model failures. The publication of AlphaFold2 revolutionised the prediction of protein structure by demonstrating heavy atom backbone structure prediction with a root mean square deviation (RMSD) of 0.96 Å, and all-atom prediction at an RMSD of 1.5 Å.^1^ However, it could not have done so without decades of high-quality, structured data made available by the Protein Data Bank (PDB).^2^ The uniform entry of data for over 200 000 protein structures was essential in providing accurate, validated entries that AlphaFold was able to train upon. Chemistry does not yet have a comparable resource.

Chemistry's experimental datasets are fragmented across journals, institutions, and proprietary repositories, collected under inconsistent conditions and formatted without common standards. As such, a direct analogue of the PDB-to-AlphaFold pipeline does not yet exist for tasks like small molecule discovery. When fragmented datasets and inconsistent data collation standards are considered in conjunction with the size of chemical space, estimated at around 10^60^ molecules in drug-like space alone,^3^ it becomes clear that organised, deliberate effort is not merely advisable but essential to making meaningful progress across chemical space.

Computational methods offer a potential solution to tackling exploration of chemical space, providing adaptable methods that can be tuned based on preferences towards accuracy and scale.^4^ Further, within a problem-defined domain, the production of computational data is systematic and scalable in ways experimental data struggles to parallel, though this scalability is necessarily targeted rather than exhaustive; even the largest contemporary databases remain many orders of magnitude smaller than the full extent of drug-like chemical space, and the value of computational data lies in directed coverage of a relevant region rather than coverage of the whole. Landmark computational datasets, from the quantum mechanical driven QM series and ANI potentials to molecular dynamics repositories, already demonstrate this in practice, providing millions of data points generated under consistent protocols within deliberately chosen, problem-defined regions of chemical space.^3,5–13^ Raw entry counts alone can understate this advantage: PubChem records more individual compounds than OMol25, 123 million against 101.7 million, but its entries are drawn from heterogeneous experimental sources and are frequently sparse in the properties relevant to a given application, whereas every OMol25 entry carries a complete, consistently calculated set of properties at a defined level of theory.^14,15^ As such, computational data is capable of providing a deliberately targeted complement to experimental data, offering a constructed resource in which accuracy and cost can be controlled to match the demands of a given application.

This perspective takes a deliberately data-centric viewpoint, arguing that the design of training data is frequently a primary constraint on chemical AI. The scale of chemical space precludes exhaustive computational coverage. The value of computational data lies instead in the ability to generate reproducible, consistently labelled data systematically and at scale within regions selected for defined applications. In this problem-defined sense, data quality, targeting, fidelity, diversity and scale jointly establish model capability and its domain of applicability.

We begin in Section 2 by examining the experimental datasets that currently underpin chemical AI and the limitations that constrain their direct use, before turning in Section 3 to the computational infrastructure that has emerged to address them. Section 4 describes hybrid and transfer learning strategies that combine the strengths of both data types, and Sections 5 and 6 present case studies and an outlook for deliberate, community-driven data design respectively.

## Experimental data: rich but irregular

Before considering what computational methods can provide, it is worth establishing precisely what experimental records do and do not contain, since the limitations of one determine the requirements of the other. Digitisation of chemistry has not only led to a rise in computational methods but also to the recording and archiving of experimental pursuits. Indeed, the PDB,^2^ as mentioned above, was critical in the success of AlphaFold which would not have been possible without the over 200 000 experimental data points extracted from the PDB.^1^ The rise of large curated reaction databases such as Reaxys and SciFinder,^16,17^ alongside open compound and bioactivity repositories like ChEMBL and PubChem,^14,18^ has enabled browsing of information such as reaction data, compounds, chemical properties, and experimental procedures drawn from a vast body of published literature. Patent literature offers a further rich, if underutilised, source. Mining tools such as Pistachio extract reaction data directly from the USPTO filings, accessing information that never passes through peer review.^19^ At the level of individual documents, automated text-extraction frameworks such as ChemDataExtractor can recover structured chemical data, including compound names, properties and conditions, directly from article text and figures, extending digitisation beyond what human curation alone could achieve.^20^

The bulk of information for these databases is drawn from existing literature, meaning that data is filtered through the publication process before it is ever captured. Methods like electronic lab notebooks (ELNs) address this by capturing experimental research at the source, providing unparalleled access to all performed research and not just that which proceeds to publication.^21^ However, there are some drawbacks to such tools. Whilst incredibly useful within an organisation, there is the issue of differing records of experimental conditions, diverging levels of detail for observations, and even different software that would make collating records uniformly effectively intractable at scale.

Beyond the practical challenges of inconsistent reporting and limited coverage, there are more fundamental limitations to the use of experimental data in machine learning. There is a natural bias skewed heavily towards publication of positive results, meaning the crucial exploratory and negative-result steps of the experimental process are largely absent from any corpus assembled from the literature.^22^ A model trained on a reaction database drawn entirely from published literature therefore learns a systematically distorted view of chemistry, one where reactions succeed and selectivity is achieved. Batch-to-batch variability compounds this, as nominally identical conditions produce inconsistent outcomes across laboratories. The consequences for model performance include learning laboratory- or instrument-specific artefacts rather than the underlying chemistry, leading to poor transferability across experimental settings.^23^ Yield prediction is a particularly illustrative example, where models frequently struggle because training data conflates multiple independent sources of variance that cannot be disentangled without richer metadata.^24,25^

This sparsity is domain specific in character. In catalysis, experimental data, for example, turnover number, is scarce and often scattered amongst different domains, making it difficult to train models that generalise across reaction conditions.^26–28^ Further, the large scale of datasets required to train accurate models is a large limitation of experimental methods where full automation is a rarity and the gathering of data by hand at the scale needed is nigh impossible.^29^ In biological systems, protein–ligand binding data is relatively rich for established drug targets but sparse for novel scaffolds, skewing model training towards already well-characterised chemical families and away from the unexplored regions where discovery is most needed.^30^

Taken together, these factors mean that experimental datasets, however expansive, are rarely ready for direct use in machine learning without significant curation effort.^31^ The uniformity problem is equally problematic. A reaction reported at room temperature in a Scottish winter laboratory and one in an Italian summer are not equivalent conditions, and such variance compounds silently as datasets scale. Computational chemistry offers a complementary response to many of these constraints by providing more uniform data across the regions of chemical space of most interest to the model.

## Computational data as a parallel infrastructure

The rise of computational chemistry over recent decades has transformed the field, driven by ever-increasing access to capable hardware, from desktop PCs to high-performance computing clusters. Computational data are not inherently rich; for example, GDB-17 contains molecular structures without associated computed properties.^8^ Computational methods can, however, generate property-labelled data at high throughput under consistent protocols, with the calculations targeted towards deliberately chosen regions of chemical or configurational space. This provides a controlled response to some of the fragmentation and heterogeneity of experimental data discussed above.

The progression of chemical datasets derived from experimental results moving to deliberately constructed archives for use in driving forward research is of central importance to this perspective. Each successive dataset was generated not to produce more data but to address a gap in the collective knowledge that has not been filled by its predecessors. A selection of relevant datasets can be seen in Table 1.

**Table 1:** Summary of discussed chemical databases and datasets

Dataset	Size	Scope	Features & theory level
GDB-11 (ref. 5 and 6)	26.4 M Structures	H, C, N, O, F	SMILES strings only
GDB-13 (ref. 7)	977 M Structures	H, C, N, O, S, Cl	SMILES strings only
GDB-17 (ref. 8)	166 B Structures	H, C, N, O, F, S, Cl, Br, I	SMILES strings only
QM7 (ref. 32)	7 K Structures	H, C, N, O, S	Structures, energies at PBE0 level
QM9 (ref. 33)	134 K Structures	H, C, N, O, F	Structures, energies, properties at B3LYP/6-31G(2df,p)
MD17 (ref. 13)	3.6 M Conformers	H, C, N, O	Structures from AIMD, energies, forces
ANI-1 (ref. 9)	20 M Conformers	H, C, N, O	Structures, energies at ωB97X/6-31G(d)
ANI-1x^10^	5 M Conformers	H, C, N, O	Structures, energies at ωB97X/6-31G(d) and ωB97X/Def2-TZVPP
ANI-1ccx^10^	500 K Conformers	H, C, N, O	Structures, energies at CCSD(T)/CBS extrapolation
ANI-2x^11^	8.9 M Conformers	H, C, N, O, F, S, Cl	Structures, energies at ωB97X/6-31G(d)
ANI-1xBB^34^	13.1 M Conformers	H, C, N, O	Structures and QM properties at B97-3C DFT level
ZINC^35,36^	55 B Structures	Commercially available compounds	Commercially available compounds with properties
PubChem^14^	123 M Compounds	Various chemicals and associated chemical and physical properties	Experimental data from diverse sources
ChEMBL^18^	2.5 M Compounds	Bioactive drug-like molecules	Bioactivity data of drug-like molecules
Transition1x^37^	9.6 M Compounds	H, C, N, O	Reactive dataset of organic reaction intermediate structures at ωB97X/6-31G(d)
tmQM^38^	86 k Compounds	3d, 4d, and 5d transition metal Compounds	Structures and properties at TPSSh-D3BJ/Def2-SVP
SPICE^39^	1.1 M Compounds	Drug-like molecules and peptides	Energies and forces at ωB97M-D3BJ/Def2-TZVPPD
QMugs^40^	2 M Conformers	Large drug-like molecules	Varied molecular properties calculated at GFN2-xTB and ωB97M-D3BJ/Def2-SVP
MD22 (ref. 41)	223 k Geometries	MD trajectories of 7 systems from four classes of biomolecules and supramolecules	Potential energies and forces calculated at PBE + MBD
OMol25 (ref. 15)	101.7 M Compounds	Molecules spanning 83 elements	Extensive chemical properties calculated at ωB97M-V/Def2-TZVPD

The Chemical Space Project exemplifies systematic coverage by iteratively producing datasets containing molecules of up to 11, 13 and 17 heavy atoms. Published as the general database (GDB) series, the GDB-11, GDB-13 and GDB-17 contain 26 million, 977 million and 166 billion structures respectively.^3,5–8^ Produced *via* building different graph structures of varying sizes and then replacing graph nodes with different atoms, the datasets, whilst providing exceedingly useful data, highlight the sheer scale of exhaustively exploring chemical space. Interestingly, the development of the GDB-17 was driven by the desire to sample chemical space that could contain drug-like molecules rather than drug-like fragments from its predecessors. After production it was found that molecules in the GDB-17 contained mostly non-aromatic structures and provided a richer variety of molecular scaffolds than the achiral or aromatic types found in databases like PubChem.^8^

The development of GDB-17 required the use of 100 000 CPU hours even when the dataset only contains SMILES strings and no other chemical properties. However, this would be remedied in part by the production of datasets like QM7 and QM9, which contain quantum mechanical information, like optimised geometries, energies, and forces, for all the structures in GDB up to 7 and 9 heavy atoms.^32,33^ Including 134 000 structures computed with DFT at the B3LYP/6-31G(2df,p) level of theory, the QM9 dataset has proved instrumental in the training of ML potentials, capable of achieving mean absolute errors of just 0.26 kcal mol^−1^ for energy predictions.^42^

Further datasets such as the ANI series (ANI-1, ANI-1x, ANI-1ccx and ANI-2x) and PubChemQC provide access to the structure of millions of small organic molecules with DFT properties, further enabling training of accurate ML models which in turn can alleviate computational cost in future studies.^9–11,43^ Beyond small molecules, the Open Catalyst (OC) datasets, OC20 and OC22,^44,45^ provide DFT-computed structures for catalyst configurations across a wide range of surface compositions, extending the computational infrastructure to heterogeneous catalysis and surface science whilst enabling the training of models capable of screening catalytic activity at a scale intractable for direct DFT calculation.

Equilibrium-geometry datasets like QM9, whilst foundational, do not capture the off-equilibrium configurations sampled during molecular dynamics simulations. The MD17 dataset addressed this directly, providing molecular dynamics trajectories evaluated with DFT at each step for eight small organic molecules and enabling training of potentials that describe atomic motion rather than static minima alone.^13^ ISO17 extended this to isomerisation pathways, offering reaction coordinate data essential for modelling chemical reactivity beyond the equilibrium basin.^12^ Transition state databases built from nudged-elastic-band calculations further expand the accessible region of the potential energy surface, providing the rare but critical data points that govern reaction rates and selectivity.^37^

Collectively, these datasets illustrate the broader principle of computational data generation offering a level of control, and a practical advantage in reproducibility. An independent attempt to regenerate a computational dataset may not produce an identical result, but the protocol behind it is fully specifiable and rerunnable in a way that an experimental campaign rarely is. Computational data tends to be much more uniform as it is ideally generated all at a consistent level of theory with identical protocols, for example the same temperature, pressure, and pure solvent, enabling certainty in the production and source of any data generated. It is worth noting that this uniformity exists within individual datasets rather than across the computational landscape as a whole, since different datasets are routinely generated at different levels of theory to suit different applications. The FAIR principles discussed later in this perspective are intended to make this diversity navigable, rather than to remove it. The existence of multiple well defined operable datasets further strengthens the argument that targeted computational data generation can directly determine predictive and generative model capability.

As exploration of space grows larger, it becomes more difficult to exhaustively cover every molecule, sequence or property. Despite only increasing by 2 heavy atoms from GDB-11 to GDB-13 and by four heavy atoms from GDB-13 to GDB-17, there is an approximate 37 and 170 times increase respectively. This is equivalent to going from calculating the structure of one molecule requiring covering 1 mm of distance to circumnavigating the globe four times to determine all the molecules in the GDB-17 dataset, Fig. 1. Further, this shows that exhaustive search of chemical space is simply infeasible with current means, a point which can readily be understood by examining a decapeptide sequence space which contains 20^10^, or roughly ten trillion, possible sequences. A dataset of one million structures covers less than 0.00001% of this space, meaning that any model trained on it must generalise across vast un-sampled regions. Given this unavoidable sparsity, it becomes critical that every computed data point is maximally informative for the training of the ML model. This requirement is not unique to computational approaches, since experimental campaigns face the same imperative to extract maximum value from each measurement. What distinguishes computational data generation is the ability to explicitly define the data required to train the model. Recovering all relevant properties simultaneously from a single calculation, as computational methods allow, ensures that no data point is wasted through either incomplete or redundant labelling, a guarantee that experimental records rarely provide. Further, the ability to discern which calculations would return the best data for a desired outcome is of paramount importance as despite the performance of modern computing it is infeasible to calculate an exhaustive period of chemical space as was discussed above.

**Fig. 1:**
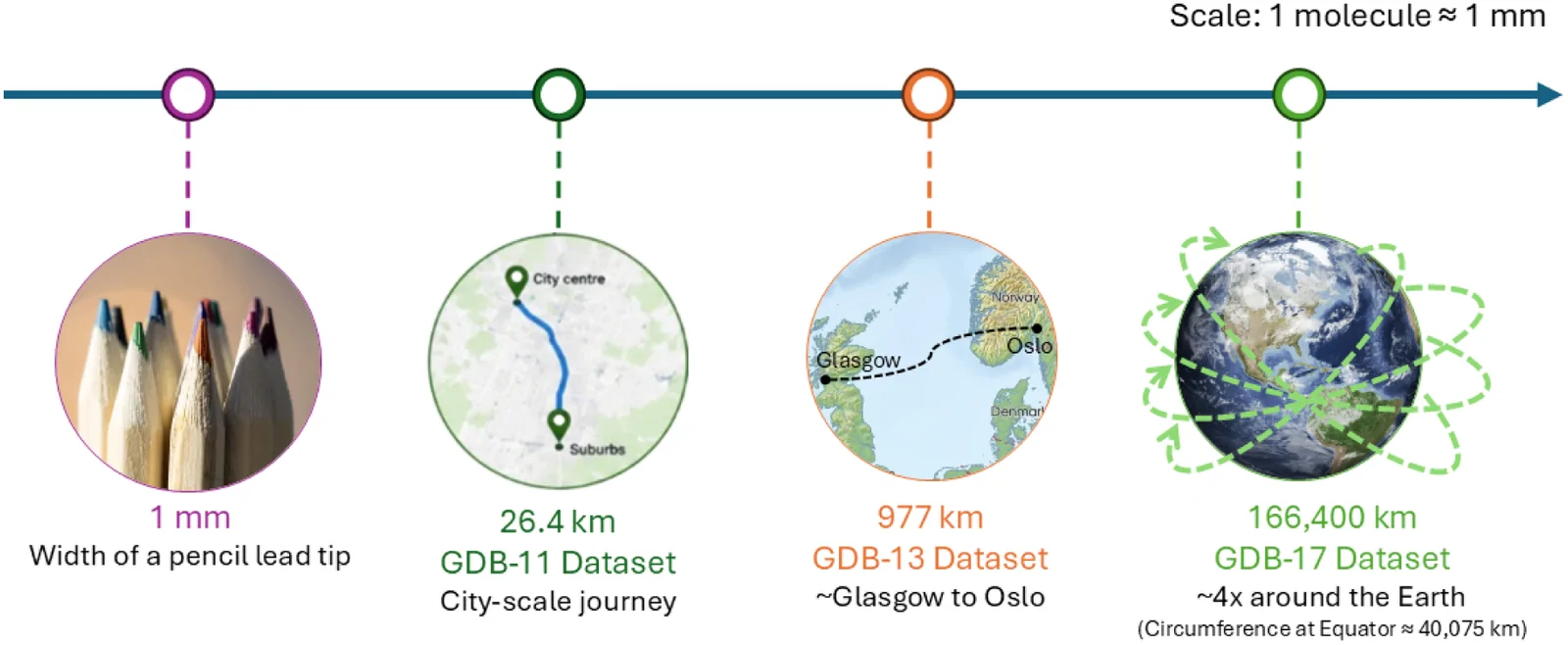
The scale of the GDB-11, GDB-13 and GDB-17 datasets shown as distances. Scale = 1 molecule ≈ 1 mm.

The availability and portability of data is of utmost importance in its application. Community repositories such as NOMAD,^46^ QCArchive,^47^ and the Materials Project^48^ partially address this by providing persistent, openly accessible storage for computational datasets, and their role in sustainable data infrastructure is discussed further in Section 6.

A related but distinct challenge is the physical portability of data once accessed. Datasets are becoming increasingly large in file size, as shown with the full OMol25 dataset weighing in at 456 GB for just over 101 million data entries.^15^ If datasets continue to grow in size then available storage can potentially become more of an issue. This is of particular concern where researchers require only a domain-specific subset of a dataset. Without modular access or openly available code, research groups are either burdened with downloading hundreds of gigabytes of data or excluded from using it entirely. Publicly available generation code resolves this by enabling groups to produce only the domain-specific subset they require, reducing storage and computational overhead whilst extending the reach of large datasets to those without dedicated data infrastructure.

In spite of the accessibility to generating datasets one of the biggest concerns, especially with the increasing impact on power grids as a result of data centres is the cost of data generation. Access to compute power has been partially alleviated by the advent of cloud computing but the cost of core hours and the time needed to generate data remain a real constraint. The OMol25 dataset, for example, required 6 billion core hours, which is beyond prohibitive for most researchers, though its open-source release upon publication means the community can draw on it immediately without bearing that generative cost.^15^

At the CCSD(T) level, scaling formally as O(N^7^) with system size, exhaustive coverage of even modest chemical spaces remains computationally intractable, necessitating the targeted sampling and multi-fidelity strategies discussed in the following section. Questions also remain about how reliably computed properties translate to experimental reality in complex environments such as biological systems, where solvent effects and surface interactions can diverge from the idealised conditions of a calculation.

Although care must be taken to balance the trade-offs when employing computational data, its application has already seen great success, particularly in complementary approaches to experimental methods, for example in mechanistic examinations.^49^ Computational data is also ideal for situations where relative trends can enable understanding even with a lack of absolute values. Further, computational methods excel in giving insight where electronic structure is concerned, *i.e.*, guiding reactivity, mechanistic investigations, and catalyst design.^50,51^ Complex conditions or environments like biological systems will often require further experimental validation, however the insight that can be gained from computational efforts can often guide or reduce unnecessary experiments.^52^ The aim is therefore not to replicate experimental reality, but to construct models that can reliably inform and predict experimental outcomes.

## Maximising the value of existing data: hybrid and transfer learning approaches

The limitations outlined above, particularly the computational cost of high-accuracy calculations and the challenge of generating data that is both broad and domain-relevant, have motivated approaches that seek to extract maximum utility from available data rather than simply generating more of it. Each of these strategies shares a common logic that rather than generating more data, they seek to make better use of the data that is already available or can be most efficiently acquired. In doing so, they shift the bottleneck from raw data volume towards intelligent data allocation, a distinction that becomes critical as the computational cost of high-accuracy calculations continues to constrain what is tractable. Active learning, transfer learning, and delta learning each represent strategies for navigating this constraint, allowing models to be trained efficiently by targeting data acquisition where it is most needed, carrying knowledge to different tasks, and learning gaps between levels of theory, rather than exhaustively sampling chemical space.

Active learning (AL) addresses the inefficiency of large dataset construction by coupling model training to targeted data acquisition or even data generation,^53,54^ and has seen a wide array of uses for data generation such as in electrocatalyst development,^55^ materials chemistry,^56^ and machine learning potentials.^57,58^ The active learning cycle, Fig. 2, shows that an initial model is trained on a small, seed dataset and evaluated against unlabelled examples. For configurations where the model is least confident, often quantified *via* ensemble disagreement (where several models disagree on the predicted value) or uncertainty estimates, high-level calculations will label the data and then are added to the training set in an iterative manner.^57,59^ This targeted sampling is particularly powerful in chemical space, where the regions of greatest relevance are often sparse and difficult to anticipate in advance, as AL concentrates computational effort precisely where the model's knowledge is weakest, enabling accurate models to be constructed at a fraction of the cost of exhaustive sampling. That the approach inevitably reflects the initial data domain is as much a feature as a limitation, guiding the model towards chemically meaningful regions rather than expending resources on configurations of little practical relevance.^57,60^ In practice, diversity sampling strategies such as farthest-point sampling or clustering in descriptor space are used alongside uncertainty-driven acquisition to ensure that successive active learning cycles do not converge prematurely on a narrow region of chemical space.^57,61–63^

**Fig. 2:**
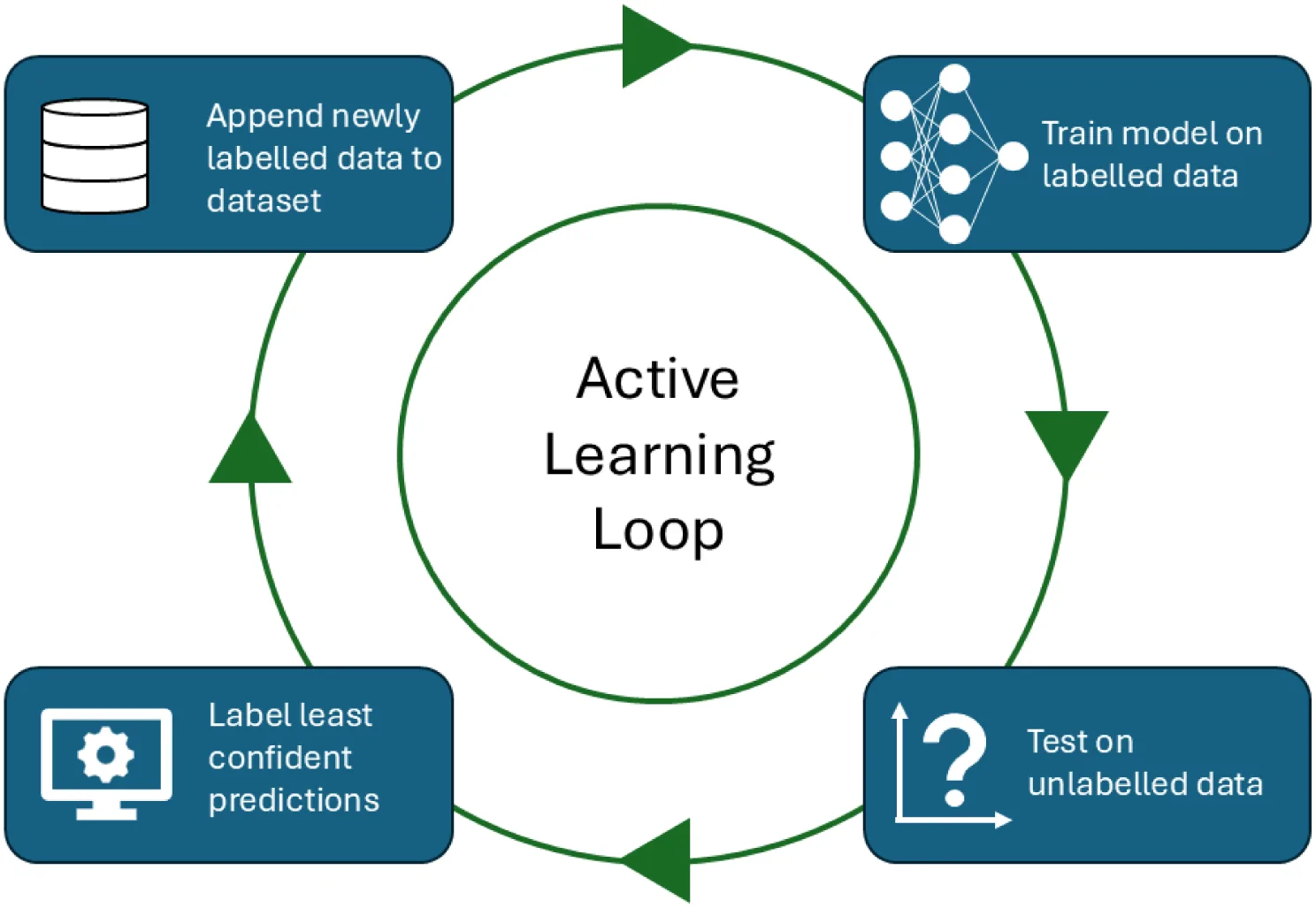
Diagram depicting the active learning cycle.

Similarly, transfer learning, Fig. 3, exploits a related insight in that general chemical knowledge encoded within a model trained on a large, lower-accuracy dataset provides a strong foundation for fine-tuning on a smaller, higher-accuracy dataset.^64^ Vermeire and Green used transfer learning to refine a model trained on quantum mechanical data with a smaller experimental dataset for prediction of solvation free energies.^65^ The produced models showed improved performance against test set data and against out-of-sample data. The value of the large initial dataset is therefore not its accuracy but its coverage. It encodes broad structural intuition that cannot be efficiently learned from the small high-accuracy set alone. Transfer learning thereby makes high-accuracy quantum chemical data tractable by ensuring that expensive calculations are used only to correct and specialise an already well-informed model. Indeed, such methods have seen further use in areas of chemistry such as NMR chemical shift prediction and more broadly in drug discovery.^66,67^

**Fig. 3:**
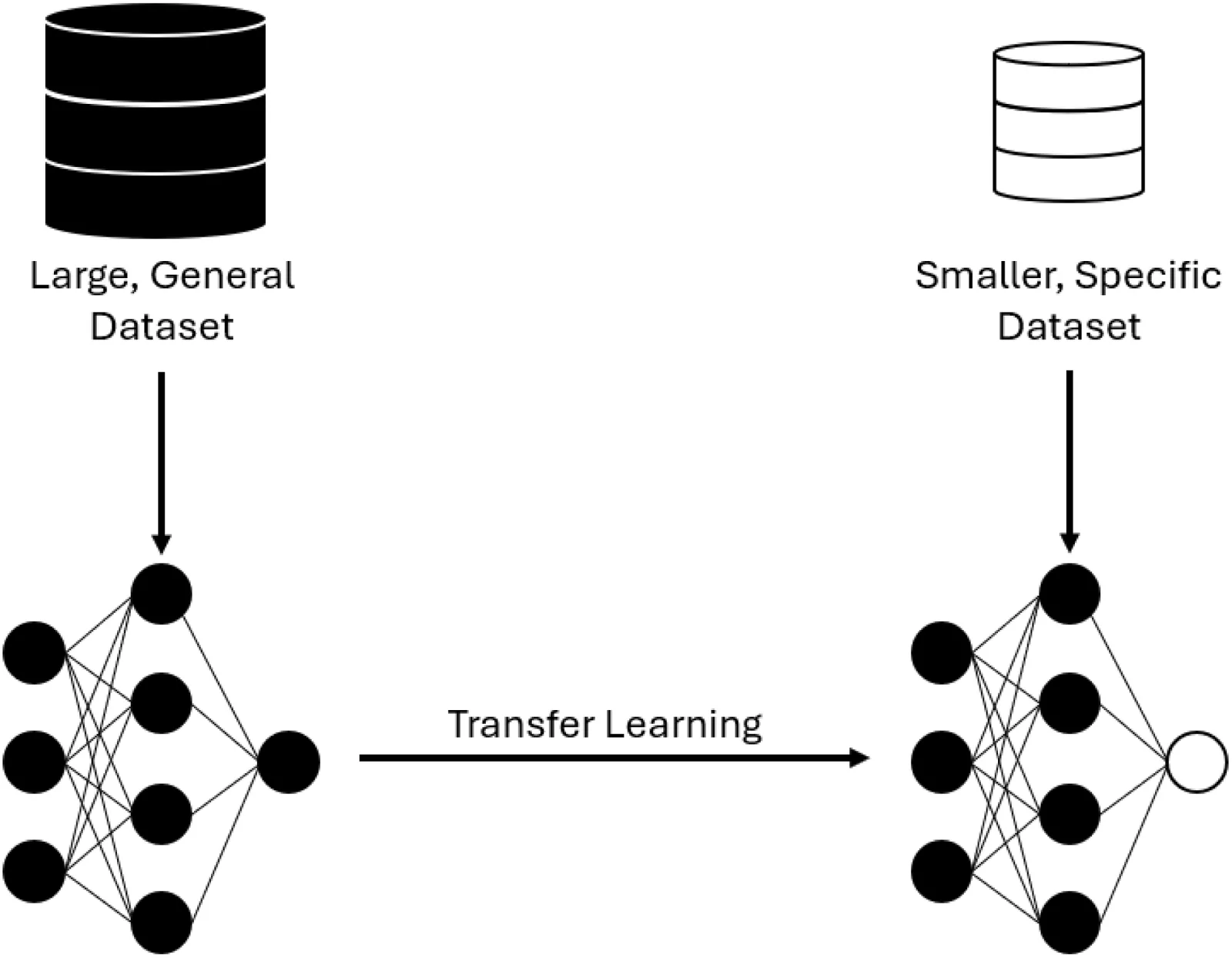
Diagram showing a generic transfer learning process.

Delta learning takes a complementary approach by learning the correction between two levels of theory rather than an absolute property. A cheap baseline method, such as a semi-empirical calculation, provides a fast approximate answer, and a machine learning model is trained to reproduce the systematic difference between this baseline and a higher level of theory,^68^ as shown in Fig. 4. The correction is then applied at the cost of the baseline method, providing access to higher-accuracy predictions without the corresponding computational burden. This was successfully demonstrated by Ramakrishnan *et al.*, who developed a set of delta learning models to correct semi-empirical and DFT thermochemical properties and electron correlation energies to highly accurate post–Hartree Fock methods at near chemical accuracy (sub 1 kcal mol^−1^) at a fraction of the cost.^69^ Other areas of delta learning include prediction of the difference between CCSD and CCSD(T) energies and prediction of pharmacokinetic properties.^70,71^

**Fig. 4:**
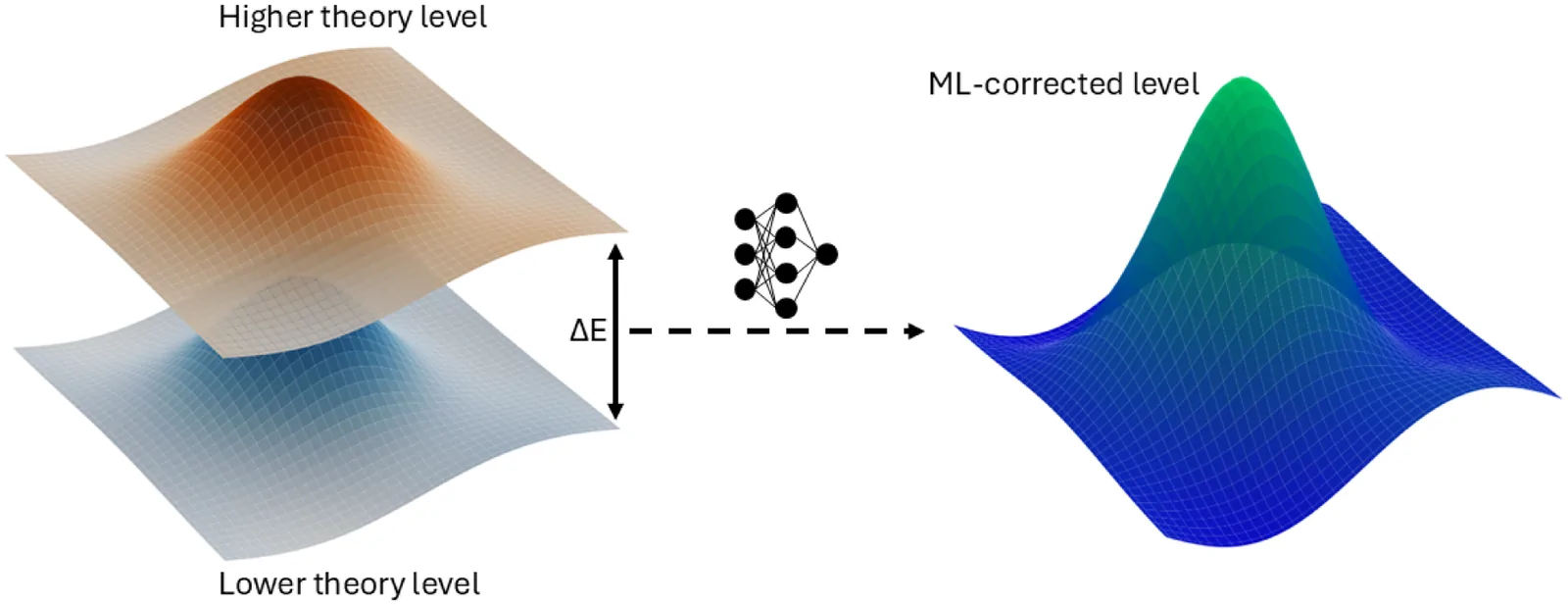
Diagram depicting the delta learning process between two reference theory levels (orange and blue) and a new correction level (blue/green).

A further consideration in hybrid approaches is the principled handling of uncertainty. Combining experimental and computational data introduces two distinct sources of variance that must not be combined. These are noise that is intrinsic to the data, whether from measurement error in experiment or numerical convergence in computation, and uncertainty arising from gaps in the model's training distribution.^60^ Computational data, generated under uniform protocols, provides a low-noise baseline that can be used to calibrate model uncertainty estimates, distinguishing genuine model ignorance from data quality issues. Ensemble methods, in which multiple models trained on overlapping subsets of the data produce a distribution of predictions, offer a practical approach to uncertainty quantification in this mixed-fidelity setting, and their disagreement serves as a direct signal for active learning cycles to acquire new, targeted data.^57^

Each of these strategies becomes most powerful when experimental and computational data are integrated rather than treated as separate resources. A compelling demonstration of this is found in the prediction of site selectivity in iridium-catalysed C–H borylation reactions, where experimental data was combined with DFT and semi-empirical calculations *via* a delta learning framework, producing a model capable of generalising site selectivity predictions accurately across previously unseen substrate classes.^72^ Similarly, 2D structures, drawn from the Cambridge Crystallographic Data Centre (CCDC),^73^ have been used as the basis for a transfer learning model proficient in predicting toxicity, synthetic yield, and olfactory character across structurally diverse chemical space.^74^ The breadth of prediction targets, spanning toxicology, synthetic accessibility, and sensory properties, illustrates that the value of transfer learning lies not only in the quality of the source data but in the generality of the structural features it encodes. The CCDC, by virtue of covering more than 1.4 million compounds, provides a representation of chemical space rich enough to transfer usefully to tasks far removed from crystallography. In both cases, the key advantage is not the volume of data but its strategic combination. While experimental observations anchor the model in physical reality, computational data provides the coverage and resolution needed to generalise.

These strategies are most instructive when examined through their application to real discovery challenges. The following section considers two such challenges in detail: the development of neural network potentials, where data strategy and model capability are inseparably intertwined, and the computationally driven design of pore-forming peptides, where AL across a space of 25.6 billion sequences demonstrates that the same principles extend beyond molecular physics to biomolecular function.

## Case studies: from potentials to peptides

Machine-learning potentials (MLPs) can approach DFT-level accuracy while providing speed-ups of up to 10^6^, enabling simulations that would otherwise be intractable.^9^ This accuracy, however, holds only within or close to the region of configurational space sampled during training. Extrapolation to substantially different geometries or chemistries typically degrades performance. Data strategy and model architecture therefore jointly determine the domain within which an MLP can be trusted. Neural-network potentials learn potential-energy surfaces from reference geometries, energies and forces generated using methods such as DFT and CCSD(T); their reliability consequently depends on the fidelity, quality and coverage of the training data and on a clearly defined domain of applicability.^75^ Overall, MLPs have already demonstrated significant ability, driving microsecond-scale simulations,^76^ studies in heterogeneous catalysis,^77^ transition metal complexes,^78^ and physicochemical property prediction,^79^ but continue to have a wide and varied application space.^80,81^

This data dependency is substantial, with earlier MLPs typically requiring datasets in excess of one million data points to achieve broad chemical coverage. ANI-1, achieving sub-kcal mol^−1^ accuracy *versus* DFT for small organic molecules, required a training dataset of 17.6 million data points.^9^ ANI-1x achieved comparable accuracy using AL to reduce this requirement to approximately 5 million points, a threefold reduction attributable entirely to more principled data selection rather than any architectural change.^57^ Progress in architectural design has led to the development of equivariant message-passing networks such as NequIP and MACE,^82,83^ that encode rotational and translational symmetry directly into their structure. This provides greater data efficiency relative to earlier approaches. This is highlighted well by MACE-MP-0,^84^ a model that achieves strong performance across materials chemistry when trained on only 150 000 individual molecules drawn from the materials project,^85^ orders of magnitude smaller than the datasets underpinning the ANI series.

Transfer learning and multi-fidelity strategies offer another route to the same destination. Rather than generating large-scale datasets at coupled cluster level, potentials such as ANI-1ccx and approaches built on delta learning leverage existing DFT-level data to reach coupled-cluster accuracy at a fraction of the cost, demonstrating that strategies for navigating data constraints are inseparable from the models they produce.^86^

The reusability and accessibility of these computational datasets represent another critical infrastructure consideration. Landmark datasets such as ANI-1x,^10^ MD17,^13^ and SPICE^39^ have been made publicly available through dedicated repositories with permanent DOI links, thus enabling independent validation, transfer learning, and further research. However, many computational datasets remain siloed within individual research groups. This limits their broader impact and prevents the community from building upon existing computational efforts, requiring redundant data generation for similar areas of chemical space.

Beyond reducing dataset size, AL actively shapes the character of the data generated. By concentrating sampling on configurations where model uncertainty is highest, iterative AL cycles produce datasets that reflect the genuine complexity of the potential energy surface rather than uniform or human-biased sampling. MLPs continue to benefit from this practice in areas such as reaction mechanism exploration and heterogeneous catalysis.^34,87^

Much like negative results driving experimental chemistry, models can also benefit from the inclusion of ‘negative’ data. In experimental chemistry, knowing that a reaction fails under certain conditions is as informative as knowing that it succeeds as the failure constrains the mechanistic hypothesis. In computational terms, a training set that includes only well-behaved, near-equilibrium structures produces a model that has never encountered the boundaries of its own validity and therefore cannot recognise when it is about to leave them. For example, by including structures and energies away from equilibrium, *i.e.*, with unnaturally close atoms, models can be taught to associate such structures with enormous energies and steer their predictions towards the correct structures.^79^ It is essential that models built to generalise across large swathes of chemical space can discriminate ‘forbidden’ regions from those that lie on the appropriate potential energy surfaces. This leads to models that can predict structures, energies and forces with precision, driving accelerated workflows. This was shown with the development of DeepSolv,^79^ an ANI-like model for p*K*_a_ prediction of small molecules, shown in Fig. 5, a task that requires accurate energy prediction throughout the whole workflow. Further, the inclusion of off-equilibrium data is necessary for accurate modelling of high energy molecules^88^ and processes.^89^ It is worth noting that architectural gains, for example from ANI to MACE, only become meaningful once both data coverage and quality are high.

**Fig. 5:**
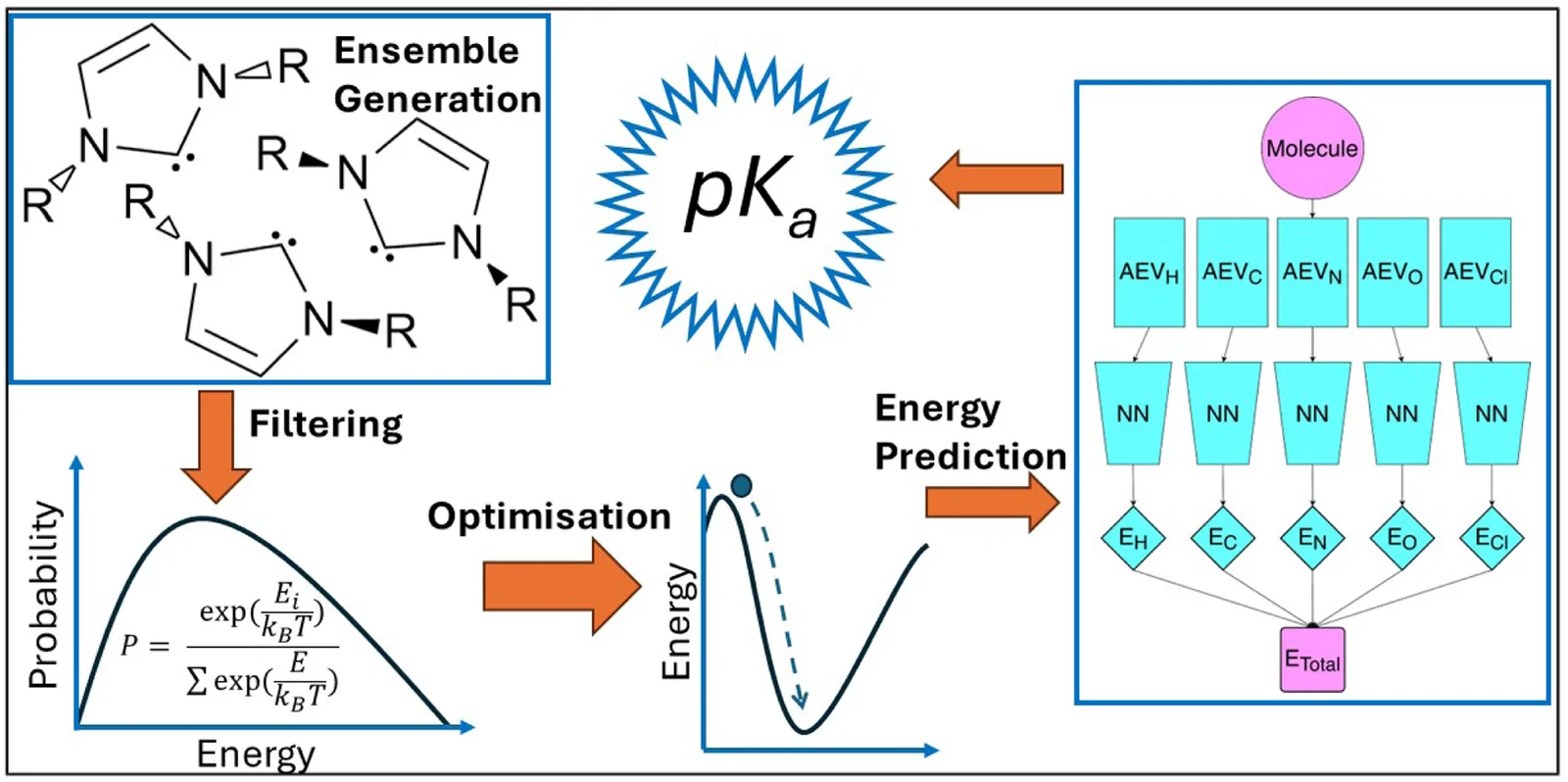
Workflow depicting the process of p*K*_a_ prediction with DeepSolv.^79^ Reproduced from ref. 79 with permission from the PCCP Owner Societies, copyright 2024.

The data strategies developed for NNPs illustrate a principle that extends well beyond molecular potentials. In each case examined above, model capability was determined not by architectural choices but by the deliberate design of the training data, and this logic translates directly to biomolecular design, where the challenge shifts from mapping potential energy surfaces to navigating sequence–function relationships across combinatorially large spaces. The search space of octapeptide sequences spans 25.6 billion structures, far exceeding experimental synthetic capacity, yet computational screening guided by AL enabled rapid identification of sequences with previously unknown pore-forming ability. Central to this approach was a discriminative metric, area-per-lipid (APL), grounded in research establishing bilayer thinning as a prerequisite to pore formation.^90^ By computing precisely the property that encodes the target behaviour, rather than accumulating a broad panel of descriptors, each simulation contributes maximally informative data with minimal redundancy.

Starting from the full octapeptide space, successive rounds of increasingly sophisticated computational screening narrowed the search to promising candidates. After seven AL cycles, the highest-performing peptides were selected for experimental screening, with additional consideration given to chemical variance across candidates to maximise the probability of identifying a hit. Together, the APL metric and iterative refinement constituted a robust, computationally driven pipeline for the discovery of novel pore-forming sequences. This process is depicted as a flow diagram in Fig. 6.

**Fig. 6:**
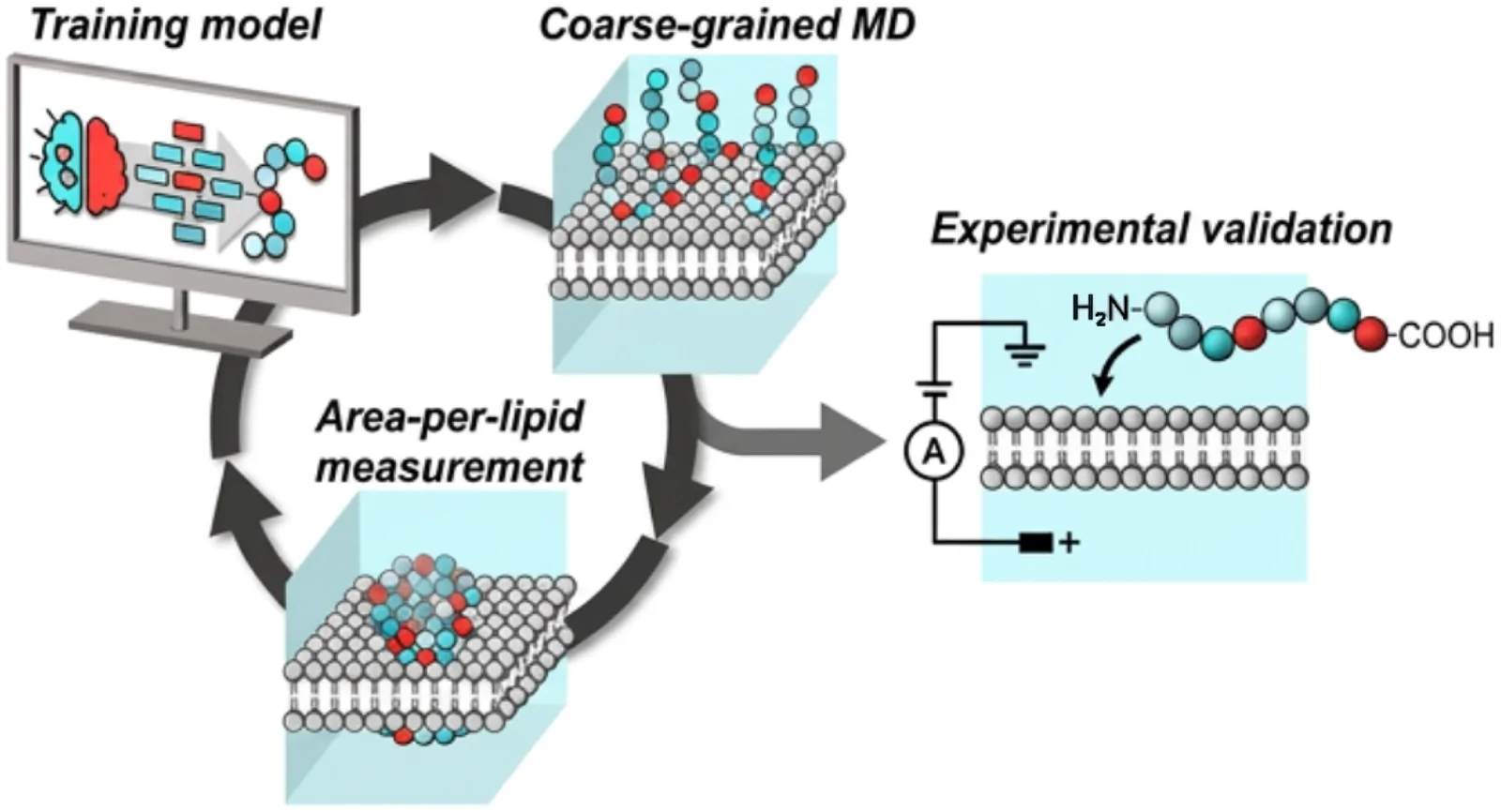
Diagram depicting the workflow of the active learning pore-forming peptide platform.^91^ Reproduced from ref. 91 with permission from the PCCP Owner Societies, copyright 2024.

Crucially, the experimental testing of predicted sequences closed the validation loop, where computational predictions guided experimental synthesis and experimental results refined the computational approach. The outcome of this work showed that the AL was capable of predicting and verifying the pore-forming ability of nine octapeptides and the first ultra-short peptides capable of membrane channel formation. This exemplifies the data-driven paradigm where dataset quality directly determines discovery success: when combined with computationally-driven screening informed by strategic data generation principles, a campaign of this scale becomes streamlined, targeted, and produces verifiable results that validate both the metrics utilised and the overall AL procedure.

Together, the studies discussed above reveal common principles underlying successful computational data generation for ML applications. These include strategic sampling through active learning, discriminative metrics that capture target properties, and the deliberate inclusion of high-energy or physically implausible configurations so that the model learns to avoid unphysical regions.^79^ Targeted sampling can reduce the amount of data required to achieve a defined level of model performance, although the necessary dataset size remains dependent on the chemical diversity, property complexity, fidelity and accuracy required. What remains is to formalise this logic across the chemical sciences by determining what data are needed and what infrastructure is required to generate, curate and share them.

## Outlook: data by design for generative chemistry

The case studies examined above suggest a trajectory, and following it to its logical conclusion demands a more deliberate approach to how computational datasets are conceived and constructed. The relevant questions are what must be calculated to enable a specific prediction, how extensively the relevant domain must be sampled, and whether the required data can be generated at tractable cost. QM7 and QM9, ANI-1 and MD17 illustrate different sampling strategies: equilibrium structures, displaced or off-equilibrium geometries, and molecular-dynamics trajectories, respectively.^9,13,32,33^ These datasets should not be regarded simply as successive increases in size; each was constructed to address a different model or data requirement. Expanding the sampled domain or increasing the fidelity of the reference data carries additional computational cost, making the balance between targeting, coverage and tractability central to dataset design.

Continued development into standardised curation of data would be most seamless when there is existing guidance to follow. The FAIR principles, first described by Wilkinson *et al.*, lay out an easy to follow set of guidelines for managing scientific data (https://www.go-fair.org/fair-principles/, accessed 20 February 2026) based on data being findable, accessible, interoperable, and reusable.^92^ Whilst these principles possess broad interpretation, they have obvious implications upon the chemical sciences.

Broad implementation of FAIR principles would allow wide adoption of datasets, models, and code without redundant calculations or extensive recoding. As datasets continue to grow, for example GDB-17's 166 billion SMILES strings or OMol25's 102 million DFT calculations,^8,15^ the ability to generate use-case-specific subsets becomes as important as the datasets themselves, directly addressing concerns around computational cost and accessibility.

The PDB demonstrates that community-wide coordination is achievable when submission, recording, and archiving standards are easy to adopt and evolve. Within chemistry, benchmarks such as MoleculeNet and MS25 enable validation of models on curated public data, allowing meaningful comparison and clearer identification of where models fail rather than merely comparing metrics.^93,94^ Benchmarks that evolve alongside current methods, rather than becoming fixed targets, will sustain this value as the field advances.

More recently, generative models have been applied to chemical problems including synthesisability-first molecule generation,^95^ and *in silico* drug discovery for complex diseases.^96^ Discriminative models answer “what properties does this molecule have?” whilst generative models must address “what molecules could have these properties?”. This requires fundamentally different dataset characteristics that not only span chemical space but encode the rules governing chemical validity, synthetic accessibility, and the distribution of molecular properties, so that the model learns to propose structures that are both novel and realistically achievable. Rather than consuming an existing dataset, generative models can become producers of new training material, and this section examines what infrastructure that role demands.^97^

Where the case studies above demonstrate how targeted, deliberately designed computational datasets shape model capability within defined domains, generative modelling introduces a setting in which experimental grounding becomes a necessary complement rather than a secondary check, for the reasons explored below. Computational data generated with generative models can be used in the training of new ML models with rapid *in silico* screening of data by methods like MLPs to label structures with energies and forces.^60^ This paradigm connects naturally to the hybrid approaches discussed in Section 5, where generative models act as a proposal engine and computational methods serve as the evaluative filter. However, there is risk involved that models trained predominantly on computational data are susceptible to issues such as hallucination and mode collapse,^98,99^ drifting towards regions of chemical space that satisfy learned statistical patterns but bear no resemblance to synthesisable or physically meaningful compounds.^100^ Experimental grounding therefore remains essential, anchoring the generative cycle to the reality of measurable chemistry and preventing the compounding of errors that purely *in silico* loops can introduce.^97,99^

The generative paradigm introduces a further risk that extends beyond mode collapse. As models begin producing new structures from datasets like QM9 and OMol25, the widespread use of these same datasets for training creates conditions for leakage, where structural similarity between generated and training molecules means apparent generalisation reflects memorisation rather than genuine chemical insight.^15,33^ This echoes the need for curated benchmarks like MoleculeNet mentioned above.^93^ Additionally, to better direct efforts in the exploration of chemical space, the importance of realising how much data is enough cannot be overstated. It is difficult to speculate on whether the correct answer lies in whether sufficiently large and diverse chemical datasets could enable foundation models for chemistry similar to GPT-series models or whether the diversity of chemical data precludes a single unified model. The point of diminishing returns where additional data does not positively impact upon model performance should be a point of further study, particularly when the scale of chemical space is considered.

In part, the challenges facing computational chemistry are those that, when addressed, will further the efforts towards unified models addressing multiple types of chemistry. Currently, many datasets are singular in nature, and models trained upon such datasets then lack any of the cross-linking that routinely takes place in laboratories, *e.g.*, the linking of synthesis with spectra and other characterisation techniques. This presents an opportunity for multi-modal models that could enable generative models that query multi-disciplinary databases simultaneously providing a more grounded approach to prediction. Early steps in this direction already exist, where models that jointly embed molecular structure and NMR spectra have demonstrated the ability to predict structural assignments from spectral data alone, and systems that integrate synthetic route information with property predictions have shown promise for drug candidate screening.^95,101^

Multi-modal integration is most consequential when applied to inverse design.^102^ The challenge of specifying desired properties and generating structures to match them is a less mature problem area than property prediction from structure. Although efforts in materials design and transition metal complex optimisation are already underway, there remains room for continued development.^103,104^ Generative models require careful consideration of training data with additional detail to the type of data and desired outcome.^105^ This links directly with thoughts echoed earlier regarding the potential sparsity of chemical datasets skewing exploration towards already well-characterised chemical families. Coupling new models to retrosynthetic tools and reaction databases will enable the design of synthetically tractable compounds. Overall, multi-objective inverse design that would account for activity, selectivity, and ease of synthesis will require richer, consistently labelled datasets for training data than those currently available.

As dataset development matures and the infrastructure to support it becomes more prevalent, the foundation for targeted, deliberate AI models will strengthen accordingly. Practices such as subset generation further position computational data not as a secondary resource to experiment, but as a targeted, deliberately designed one that can determine model development. Realising this requires a systematic collaboration between researchers worldwide, adoption of frameworks, like FAIR, ensuring that computational data is generated where it will be most useful.

## Author contributions

Ross Urquhart: investigation, writing – original draft, writing – review and editing. Tell Tuttle: writing – review and editing, supervision.

## Conflicts of interest

There are no conflicts to declare.

## Data Availability

No primary research results, software or code have been included and no new data were generated or analysed as part of this review.
